# An Outline of the History and Treatment of the Fever of Growth

**Published:** 1836-07

**Authors:** 


Art. XIV.—*Das Streckfieber und dessen Behandlung, im Umriss dargestellt.* Von Dr. G. C. Reich, Professor der Medicin, &c.—*Berlin,* 1835.

*An Outline of the History and Treatment of the Fever of Growth.* By Dr. G. C. Reich, Professor of Medicine, &c.—*Berlin,* 1835.

In German scientific writing we have often to forgive the clumsiness of the form for the sake of the importance of the matter. Valuable discoveries are frequently wrapped up in a phraseology which stoutly resists our first attempts to penetrate its meaning. But we readily pardon our author the trouble he may have caused us if we find any thing in him to requite it. There is even a zest in earning information, which one does not feel in receiving it without exertion. But, in the present case, we cannot say that the labour which it costs us to understand our author is fully rewarded. Dr. Reich commences with a philosophical introduction which is but feebly connected with his professed object; he propounds some important statements, but without taking the trouble to go into either proof or elucidation; and terminates the book before properly entering upon the subject.

His positions are as follows:—

In the first place, that there are five stages of growth, or, as he calls it, of *evolution ;* viz. *teething, the changing of the teeth, puberty,* and *the period between puberty and manhood :* he is rather doubtful at first about the existence of a fifth stage, but, finally, concludes that the period at which *the wise tooth* makes its appearance ought to be considered as such. An increased flow of blood to the part where the phenomenon of growth or *evolution* takes place is required to effect that phenomenon. The author makes use of the words orgasm, turgescence, and congestion, in order to describe the condition of such a part. During the first two stages of growth, this condition is found in the head; during the third stage, it is found in the organs of generation and in those of sense; and, during the fourth, principally in the lungs.

His second position is, that this congestion is frequently attended by symptoms, (which he denominates in the aggregate "*the fever of growth;*") and which symptoms are, increased heat and redness of particular parts of the face; sometimes intermitting chilliness and paleness of the lips, nose, and cheeks; peevishness, caprice; pain in the head; disagreeable breath; chattering of the teeth; indisposition to eat and drink; vomiting; flying pains in the limbs; unsteady gait.

These are, doubtless, pathological signs, but they are never to be considered as a real disease, and never to be treated antiphlogistically. The reason of this we shall find in his third position. It is that the congestion or turgescence above mentioned is not of an inflammatory nature, never tends to inflammation, and never terminates in it, except in consequence of improper treatment. Therefore, in the first two stages of growth, cerebral congestion, which is a provision of nature to effect the phenomena of *evolution,* is never to be counteracted by depletion. Bloodletting is especially contraindicated. No fear need be entertained of inflammation; *the author never saw a case of acute inflammation of the brain in a child!* The latter disease (if it terminates fatally,) supposes the deposition of plastic matter between the membranes; whereas, in children, one finds nothing except an extravasation of watery and albuminous fluid in the ventricles. Dr. Reich holds, then, that the extravasation of an albuminous fluid is not a sign of inflammation.

He gives the following curious account of the manner in which antiphlogistic treatment may cause a normal congestion to produce extravasation of a watery fluid in the ventricles of the brain. Supposing the child to have been bled, and nourishment to have been denied it, he describes the absorbents as having a treble duty to perform. They have first, to find matter for the nutrition of the body; then, for its growth; and, finally, to supply the place of that of which it has been deprived by the physician. All this matter must be found in the body of the child itself, seeing that the antiphlogistic treatment precludes all nourishment from without. An unhealthy activity of the absorbents is consequently set up. All the vessels which lead to the heart are loaded with reabsorbed matter. In the brain a normal congestion is superseded by an anormal one; extravasation takes place in the ventricles; the physician thinks he has to deal with a case of acute inflammation, and bleeds till the patient dies. This is nearly tantamount to the assertion that bleeding promotes plethora.

In the third stage of growth, our author asserts that the organs of sense present symptoms of congestion, which are only aggravated by the antiphlogistic treatment. He very seldom descends to particulars: he instances, however, the junction of the *os annulare* with the temporal bone, and the consolidation of the *pars squamosa* with the *pars petrosa*, as giving rise to symptoms which simulate inflammation, but which ought never to be treated as such.

These several congestions, should they proceed to an extreme, may be controlled by cold applications. This is the utmost extent to which the author's theory allows him to interfere, except in affections of the lungs, during the third stage of growth, where there often exists a disposition to inflammation, which ought to be vigorously opposed. But he is continually asserting that the antiphlogistic treatment, and more particularly the exhibition of calomel in the case of children, are extremely prejudicial, inasmuch as they render a healthy congestion morbid, and violently interrupt the processes of nutrition and growth which are essential to the health of the child. This is the part of his book which is most worthy of consideration: unfortunately, it is neither illustrated by observation nor supported by proof.

In treating of the exhibition of calomel to children, he alludes to the English, and mentions Quin and Dobson as authors whose pernicious recommendations ought to be most carefully avoided.

He boasts of a treatment, he tells us, peculiarly simple and easy: it assists, instead of perverting, the course of nature. We have mentioned already his cold applications, which are to regulate but not subdue congestion. Of course, the patient is to breathe a cool and salubrious atmosphere; but Dr. Reich's main remedy is *muriatic acid.* This is applicable to the fever of growth, in whatever stage it may make its appearance. He forgets to mention the exact dose; but he can confidently recommend it in all those cases where bad management on the part of the nurse, and bad treatment on the part of the physician, have not succeeded in imparting to the congestion a more or less inflammatory type.

